# Does Telescoping Exist in Male and Female Gamblers? Does It Matter?

**DOI:** 10.3389/fpsyg.2017.01510

**Published:** 2017-09-05

**Authors:** Yasmin Zakiniaeiz, Kelly P. Cosgrove, Carolyn M. Mazure, Marc N. Potenza

**Affiliations:** ^1^Interdepartmental Neuroscience Program, Yale University School of Medicine New Haven, CT, United States; ^2^Department of Radiology and Biomedical Imaging, Yale University School of Medicine New Haven, CT, United States; ^3^Department of Psychiatry, Yale University School of Medicine New Haven, CT, United States; ^4^Department of Neuroscience, Yale University School of Medicine New Haven, CT, United States; ^5^Women's Health Research at Yale New Haven, CT, United States; ^6^Child Study Center, Yale University School of Medicine New Haven, CT, United States; ^7^National Center on Addiction and Substance Abuse, Yale University School of Medicine New Haven, CT, United States; ^8^Connecticut Mental Health Center New Haven, CT, United States

**Keywords:** gambling disorder, gender, sex, telescoping, prevention, treatment

## Introduction

Sex/gender differences (hereafter termed “gender-related differences,” with the understanding of the American Psychological Association's (2012) definitions of sex as a person's biological status such as male or female, and gender as the attitudes, feelings, and behaviors associated with biological sex) in gambling disorder (GD) have received relatively little investigation. Existing studies have shown that gender-related differences may exist in forms of gambling preferred and performed, disorder onset, co-occurring disorders and disorder progression, especially telescoping. Given existing data, we opine that understanding gender-related differences in telescoping, as well as other gender- and gambling-related phenomena, is critical for optimizing prevention and treatment strategies. With a focus on telescoping, this piece aims to opine on the importance of studying gender-related differences to better understand telescoping and for informing effective policy, prevention, and treatment efforts.

## Telescoping

Several studies have found that women show a later initial engagement with GD but tend to progress more rapidly than men in developing an addictive disorder, a gender-related phenomenon known as telescoping (Potenza et al., 2001; Ladd and Petry, 2002; Tavares et al., 2003; Nelson et al., 2006; Grant et al., 2009, 2012c). Although the cause of the telescoping effect is not known, co-occurring disorders related to anxiety in women and alcohol use in men may not be as central as was previously hypothesized (Tavares et al., 2003; Odlaug et al., 2011; Grant et al., 2012c).

While most studies report that GD in women compared to men is “telescoped,” one study involving a large Australian twin cohort of over 4,600 individuals found no evidence of this effect (Slutske et al., 2015). In fact, this study showed that men initiated gambling at an earlier age and progressed more rapidly to a GD diagnosis than did women. It is important to consider that these findings were based on “moderately reliable” retrospective reports and secondary analyses of a sample that did not require an elaborate twin study design. Further, the results of this study may not generalize to gamblers in countries outside of Australia.

The discrepancy between the Slutske et al. (2015) telescoping finding and previous studies may be explained by several possible confounds regarding the nature of the samples. First, the age of gambling onset varied, where Slutske and colleagues reported the average age of onset to be 17–18 years for both men and women while other studies reported the average ages of onset to be 20–22 years for men and 30–34 years for women. Second, the racial and regional compositions of the samples varied between the Slutske et al. study (Australian, Northern European/Caucasian) and the other studies (North and South American, including all ethnicities). Third, the Slutske et al. study used a community-based as opposed to a treatment-based sample, suggesting that gender-related differences in GD, such as telescoping, may not hold in non-clinical populations. Future studies should investigate these and other possibilities.

## Possible explanations for telescoping

### Gambling type

Differences in performed forms of gambling by women compared to men, relating to non-strategic vs. strategic forms (Potenza et al., 2006; Grant et al., 2012b), may partially explain the telescoping effect (Potenza et al., 2001; Tavares et al., 2003). It has been hypothesized that the non-strategic forms of gambling (like slot machines) preferred and performed by women may be more addictive because the time between placing the bet and the outcome is often shorter than some other forms of gambling (Potenza et al., 2001; Tavares et al., 2003; Dowling et al., 2005). This faster display of results may in part explain why some individuals continue gambling as they may feel that they are just a short amount of time away from winning (Dowling et al., 2005). However, this point has been debated (Nower and Blaszczynski, 2010). Specific preference for forms of gambling such as electronic bingo has been related to the telescoping effect (Tavares et al., 2003); however, when type of gambling was considered in models, the telescoping effect still remained (Grant et al., 2012c), suggesting the type of gambling may not account substantially for the telescoping effect.

### Co-occurring disorders

Links between psychopathology and problem-gambling severity appear stronger in women than in men for major depression and stronger in men than in women for alcohol dependence (Lesieur et al., 1986; Welte et al., 2001; Petry et al., 2005; Blanco et al., 2006; Desai and Potenza, 2008). Women compared to men are more likely to seek treatment for GD, which may in part relate to co-occurring psychiatric disorders, particularly depression and anxiety disorders (Potenza et al., 2001; Martins et al., 2004; Desai and Potenza, 2008; Husky et al., 2015). Co-occurring anxiety, depression, and alcohol dependence with GD may exacerbate gambling-related symptoms across both genders (Parhami et al., 2014; Quigley et al., 2015). However, some studies have found that co-occurring disorders relating to depression and anxiety in women and alcohol dependence in men did not explain gambling progression (Tavares et al., 2003; Odlaug et al., 2011; Grant et al., 2012c). Among recreational gamblers, moderate/high alcohol consumption was associated with heavier gambling in men than in women (Desai et al., 2006). In adolescence, gambling behavior is associated with elevated rates of alcohol use in both boys and girl and depression/dysphoria in only girls (Desai et al., 2005). Negative mood states and depression may increase gambling-related behaviors, particularly in women (Tschibelu and Elman, 2010), consistent with negative reinforcement theories in substance-use disorders. Thus, additional research is needed to determine whether and how co-occurring psychiatric disorders may relate to telescoping.

### Biological differences

With respect to genetics, most genetic studies of GD have focused on candidate genes, such as those coding for dopamine receptors and the dopamine transporter; however, these have yielded conflicting results and have under-represented women. One candidate gene study investigating the catechol-O-methyltransferase gene (*COMT*), a gene that codes for an enzyme regulating dopamine in the prefrontal cortex and has a commonly occurring allelic variation (Val158Met) linked to enzymatic function, found that individuals with Met/Met genotypes were twice as likely to be at-least-at-risk gamblers than Val carriers; no gender-related differences were observed (Guillot et al., 2015). Similarly, Met/Met individuals performed worse on decision-making/cognitive impulsivity tasks than Val carriers (Malloy-Diniz et al., 2013).

Using the all-male United States Vietnam Era Twin Registry, percentages of lifetime GD diagnosis were higher in monozygotic (23%) as compared to dizygotic (10%) co-twins (Eisen et al., 1998). Using the Australian Twin Registry, it has been estimated that genetic risk factors account for approximately half of the variability in GD etiology in both men and women (Slutske et al., 2010). Further, the association between GD and alcohol-use disorder has been linked in part to overlapping genetic risk, with men showing a higher correlation between the two disorders than woman when using dimensional (but not categorical) measures (Slutske et al., 2013). A recent study also found that a polygenic risk score for alcohol dependence was associated with GD (Lang et al., 2016). More research is needed to identify gender-related genetic and environmental factors linked to GD susceptibility and progression, particularly as environmental factors appear more closely linked to age of gambling onset in women as compared with men (Richmond-Rakerd et al., 2014).

In relation to neurocognitive measures, male gamblers tend to score higher on risk-taking measures than do female gamblers (Johansson et al., 2009); however, no gender-related differences were found on measures of impulsivity (Grant et al., 2012a). A few neuroimaging studies have begun to investigate the neural mechanisms of GD with respect to craving-related brain regions such as the medial prefrontal and cingulate cortices (Kober et al., 2016). In this study, women with GD showed increased dorsomedial prefrontal, posterior insula, and caudate activation when viewing gambling-related videos, compared to men with GD. The extent to which such differences may relate to telescoping warrants investigation.

### Sociocultural differences

Women may have a later age of GD onset because they may have less exposure to gambling opportunities. Social norms historically have more strongly opposed gambling in women as compared to men (Potenza et al., 2001); however, this may be changing over time. It has been argued that women experience sociocultural pressures to seek treatment despite gender-related similarities in treatment severity, which may influence observations of telescoping reported in alcoholism (Piazza et al., 1989). Sociocultural differences between gender groups require further investigation to investigate how they relate to disease development and progression.

## Impact on prevention and treatment

While there is currently no evidence for gender-related differences in treatment effectiveness, the authors would like to recommend a few guidelines for more effective prevention and treatment strategies with respect to telescoping considerations. First, because women may progress to GD faster than men and boys tend to exhibit GD more so than girls, earlier interventions for both genders are advised. Second, gambling may be triggered in gender-related fashions—mood states for women and sensory stimuli for men. Women may more likely be triggered by depressive symptoms that are unrelated to the gambling itself, while men may more likely be triggered by gambling-related advertisements, billboards etc. (Grant and Kim, 2002; Martins et al., 2004). Thus, prior to or concurrent with GD treatment, women may benefit from assessment and treatment of depressive symptoms (e.g., through pharmacological approaches) while men may benefit from cognitive-behavioral therapy or other approaches to dampen responses to sensory stimuli, although this possibility is currently speculative. Additionally, policy approaches that regulate gambling advertisements may be particularly important for men. Third, because co-occurrence with alcohol dependence is higher in men than in women, men may benefit from treatment of alcohol-use disorders prior to or concurrent with GD treatment. While best practices may suggest concurrent treatment for co-occurring psychiatric disorders, more research is needed to determine whether sequential vs. concurrent treatments are optimal for co-occurring disorders in association with GD and the extent to which telescoping or other gender-related processes may inform such treatments. Fourth, stress and post-traumatic regulation in women might be helpful as a preventive measure before treatment, particularly for women given the particularly strong links between trauma and gambling problems in women and female-predominant tendencies to gamble as an escape from negative emotions (Getty et al., 2000; Petry and Steinberg, 2005; Dion et al., 2010). Fifth, in treatment-seeking gamblers, females more typically report abstinence, while males often report moderation as their end goals (Kim et al., 2016). The extent to which such differences may relate to telescoping warrants additional study. We suggest that treatment options may be improved if gender-related factors such as telescoping were more closely considered.

While some studies suggest that females may be an at-risk gambling group given more rapid progression of GD, research into telescoping and its etiology are at early stages. Importantly, understanding disease progression and gender-related differences matters if we are going to advance effectively intervention strategies. The relative deficiency of knowledge of gender-related differences in gambling behaviors and the health impacts of gambling could be addressed if future studies focused on early signs of gender-related differences, possible effects of different forms of gambling, problem-gambling severity, psychiatric comorbidity, and biological factors on the progression to disease in women and men. Performing gender-related analyses on sample populations that vary socio-demographically and clinically may promote a better understanding that may be translatable into improved prevention, treatment and policy efforts. Considering important aspects relating to environmental, individual, and contextual factors should facilitate these efforts for both females and males.

## Author contributions

YZ wrote the first draft of the manuscript and worked with all other authors, KC, CM, and MP on subsequent drafts. MP revised the manuscript critically for important intellectual content. All authors have approved this manuscript.

### Conflict of interest statement

The authors report that they have no financial conflicts of interest with respect to the content of this manuscript. MP has received financial support or compensation for the following: MP has consulted for and advised Ironwood, Lundbeck, INSYS, Shire, RiverMend Health, Opiant/Lakelight Therapeutics and Jazz Pharmaceuticals; has received research support from the NIH, Veteran's Administration, Mohegan Sun Casino, the National Center for Responsible Gaming, and Pfizer pharmaceuticals; has participated in surveys, mailings, or telephone consultations related to drug addiction, impulse control disorders, or other health topics; has consulted for legal and gambling entities on issues related to impulse control disorders; provides clinical care in the Connecticut Department of Mental Health and Addiction Services Problem Gambling Services Program; has performed grant reviews for the NIH and other agencies; has edited journals; has given academic lectures in grand rounds, CME events and other clinical or scientific venues; and has generated books or book chapters for publishers of mental health texts. The other authors declare that the research was conducted in the absence of any commercial or financial relationships that could be construed as a potential conflict of interest.
